# Deep sea explosive eruptions may be not so different from subaerial eruptions

**DOI:** 10.1038/s41598-020-63737-7

**Published:** 2020-04-21

**Authors:** Gianluca Iezzi, Gabriele Lanzafame, Lucia Mancini, Harald Behrens, Stella Tamburrino, Mattia Vallefuoco, Salvatore Passaro, Patrizio Signanini, Guido Ventura

**Affiliations:** 1 Department of Engineering and Geology, G. D’Annunzio University, Chieti, Italy; 2 National Institute of Geophysics and Volcanology, Rome, Italy; 3 Department of Biological, Geological and Environmental Sciences, University of Catania, Catania, Italy; 4 Elettra-Sincrotrone Trieste S.C.p.A., Basovizza, Trieste Italy; 5 Institute for Mineralogy, Leibniz University, Hannover, Germany; 6 Institute of Marine Sciences (ISMAR-CNR), Napoli, Italy

**Keywords:** Volcanology, Petrology

## Abstract

The dynamics of deep sea explosive eruptions, the dispersion of the pyroclasts, and how submarine eruptions differ from the subaerial ones are still poorly known due to the limited access to sea environments. Here, we analyze two ash layers representative of the proximal and distal deposits of two submarine eruptions from a 500 to 800 m deep cones of the Marsili Seamount (Italy). Fall deposits occur at a distance of more than 1.5 km from the vent, while volcanoclastic flows are close to the flanks of the cone. Ash shows textures indicative of poor magma-water interaction and a gas-rich environment. X-ray microtomography data on ash morphology and bubbles, along with gas solubility and ash dispersion models suggest 200–400 m high eruptive columns and a sea current velocity <5 cm/s. In deep sea environments, Strombolian-like eruptions are similar to the subaerial ones provided that a gas cloud occurs around the vent.

## Introduction

**Figure 1:**
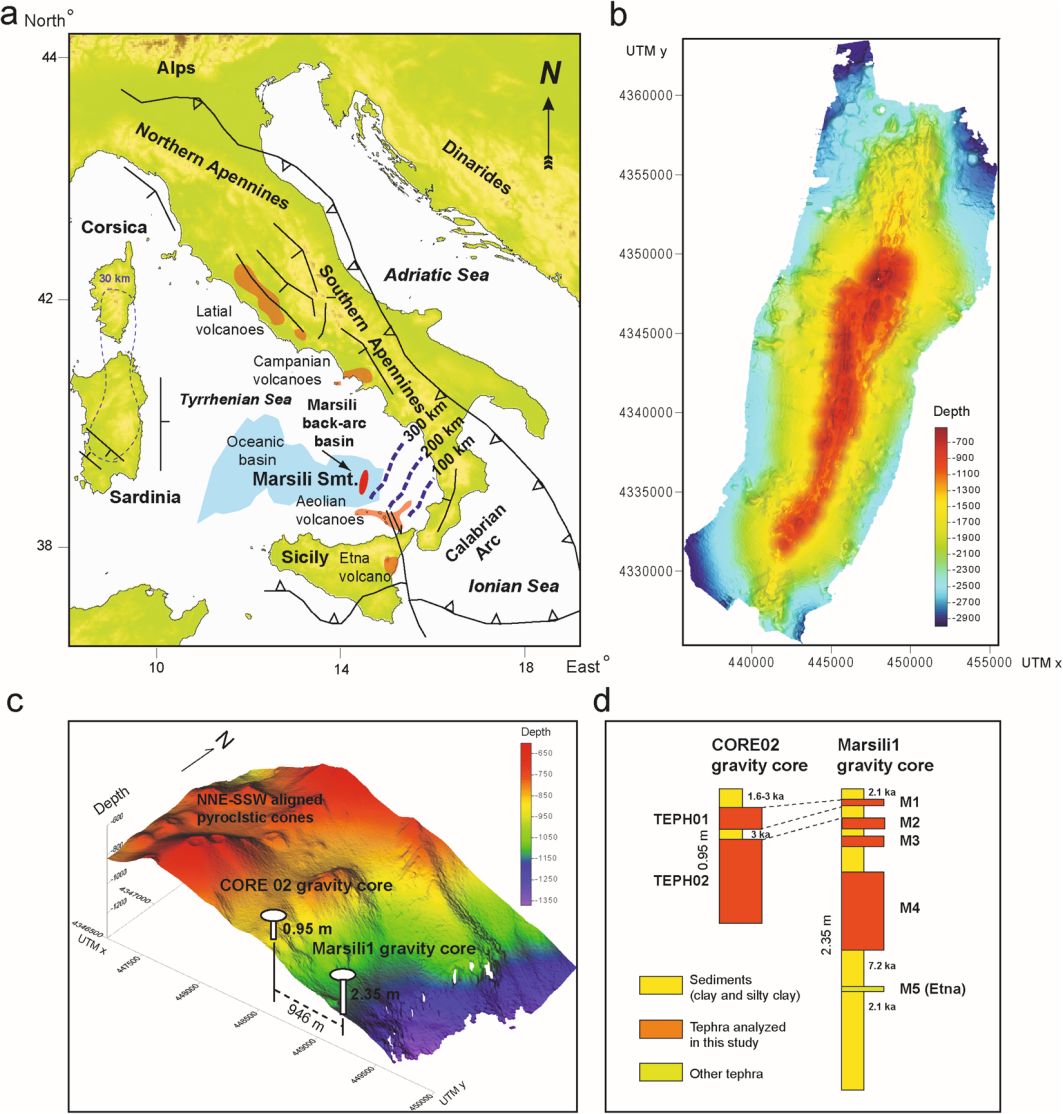
(**a**) Location and geodynamic context of the Marsili Seamount with depth of the subduction plane, and (**b**) 25 m resolution digital elevation model of the seamount. (**c**) 5 m resolution digital elevation model of the central sector of the Marsili Seamount with the location of the two cores analyzed in this study. (**d**) Schematic stratigraphy of the CORE02 and Marsili 1 gravity cores with ages (within the cautionary limit of ±0.2 ka of the ^14^C age determination) from previous studies^17,18^. The figures are generated using the software Surfer version 17.1.288 (Goldensoftware) (https://www.goldensoftware.com/products/surfer). The data in (**a**) are freely available at (https://www.emodnet-bathymetry.eu/data-products). The data in (**b,c**) are from a 5-m resolution DEM of the Marsili seamount (see Data Availability).

Two-thirds of the oceans are mantled by volcanic rocks from effusive and explosive eruptions^1^. Although deposits of deep (>500 m depth) explosive eruptions have been recognized in the last decades, their significance remains still poorly constrained due to difficulties in accessing submarine environments, lack of unambiguous distal-proximal correlations of the deposits and poor information on the vent location^2–12^. In addition, the dynamics of submarine explosive eruptions may be highly variable: observations at Mata (Tonga Trench) evidence degassing, lava flow emissions, Vulcanian- and Strombolian-like eruptions from the same vent area^11^. Our knowledge on how the deposits of this type of eruptions vary with the distance from the vent, the amount of gas in the emitted magma, the fragmentation mechanisms, the settling times of the pyroclasts, the height of volcanic plumes, and the traveling distance of the generated volcanoclastic flows is, however, poorly constrained^13,14^. Results from theoretical and analogue models show that jets and plumes can raise several hundreds of meters above vents and are, in principle, able to generate gravity currents^2–15^; however, the results of these models have not been confirmed from field data, with the possible exception represented by nowadays emerged Precambrian pyroclastic deposits^13^. Here, we present new data on tephra layers from two gravity cores collected on Marsili Seamount (hereafter MS; Southern Tyrrhenian Sea, Italy) (Fig. 1a,b). Our aim is to reconstruct the dynamics of two deep submarine eruptions occurred in historical times, analyze the spatial variability of the deposits, determine the role of magmatic and hydromagmatic processes, and clarify the physical mechanisms of emplacement of the tephra layers. The 0.95 m long CORE02 gravity core hosts two dm-thick tephra layers, while the 2.35 m long Marsili 1 gravity core contains 5 cm-thick tephra layers (Fig. 1c,d). The cores are located in the MS central sector at 839 and 943 m b.s.l., respectively. Stratigraphic, geochemical data and age determinations indicate that the two shallowest recorded tephra layers represent the proximal and distal successions of two different basaltic trachyandesitic and trachytic submarine eruptions occurred between 3 and 5 ka BP^16–18^. We characterized the three-dimensional (3D) bubble distribution (amount and shape) of these deep volcanic ashes by synchrotron X-ray computed microtomography (SR-µCT). Glass composition and dissolved volatile content of glasses are also determined and the ratio between the exsolved and dissolved gas just prior of the eruption is calculated along with the amount of water required for fragmentation. The density of the volcanic jet and the height of volcanic plume are evaluated along with the horizontal velocity (lateral expansion) of ash by reconstructing the transport dynamics. The results allow us to quantify the following relevant parameters of deep submarine eruptions: a) minimum amount of water required for fragmentation and fraction of large bubbles unrecorded in volcanic ashes, b) distance traveled by syn-eruptive ash-dominated gravity currents, c) dispersion of fall deposits, and d) height of the volcanic plume. We show that the dynamics of deep submarine eruptions may be not so different from that of subaerial eruptions. Our results have important implications for the study of recent and ancient volcanic successions, submarine eruptive mechanisms and dynamics, and tephra layers correlation analysis.

## Geological Setting and Investigated Gravity Cores

**Figure 2:**
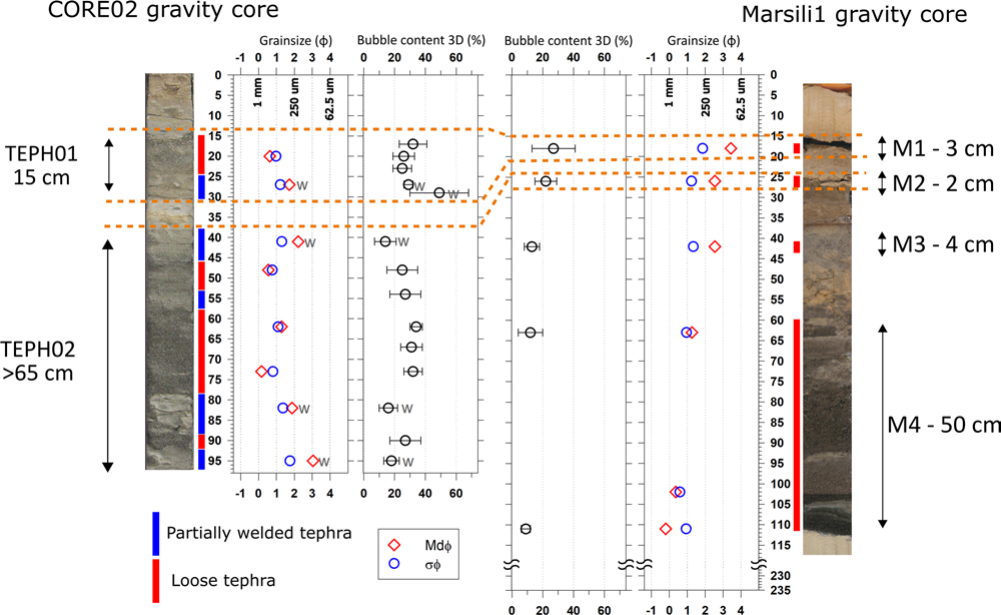
Mesoscopic stratigraphy of the CORE02 and Marsili 1 gravity cores with 2D measured grain size parameters^17,18^ and bubble content of the sub-marine tephra layers. W stands for welded. The median grain size of tephra layers in both cores is in the volcanic ash range (Mdφbetween-1 to 4), while the sorting (σφ) ranges between 0.6 and 1.9 of, i.e. between moderately well sorted to poor sorted. The gently welded ashes in the CORE02 (left) have median size and sorting lower than their corresponding loose ash levels. The median ash size in TEPH01 and TEPH02 layers is larger than that of Marsili1 (right) ash. These data are reported in Table S2.

MS (volcanic activity: 1 Ma to 2–3 ka BP) represents the axial ridge of the 2 Ma old Marsili back-arc oceanic basin, which is associated to the Calabrian Arc-Ionian Sea subduction system^19^ (Fig. 1a). MS is about 3000 m high, NNE-SSW elongated, and consists of several NNE-SSW striking dikes, small pyroclastic cones and lava flows (Fig. 1b). The tephra layers analyzed in this study come from the CORE02 (839 m b.s.l.) and Marsili1 (943 m b.s.l.) gravity cores, both located on a flat surface in the MS central sector (Fig. 1c,d). CORE02 is at a distance of about 1 km from three NNE-SSW-aligned pyroclastic cones. One of these cones is considered the source area of the tephra layers analyzed in this study^17,18^. The distance between the two recovered gravity cores is 946 m. CORE02 contains two tephra layers (15 cm thick TEPH01 and 60 cm thick TEPH02) consisting of 98–100 vol.% of volcanic ash^17^. Five tephra layers were recorded in the Marsili1 core; here, we focus on the uppermost two tephra layers, which are represented by the 3 cm thick M1 and 2 cm thick M2 ashy levels. These tephra layers represent two events of volcanic deposition occurred between 7.2 and 2.1 ka. The tephra layers recognized below M2 in the Marsili 1 core refers to the MS activity ranging from 5 to 7 ka (M3 and M4), and to a Mount Etna eruption (M5) dated about 20 ka^18^ (Figs. 1d and 2; Tables S2 and S3). According to the characterization scheme adopted for oceanic cores^20^, all the tephra layers of the two MS gravity cores represent V1- and V2-type tephra, i.e. samples with total or dominant glassy fraction. They are characterized by distinct top and bottom contacts. The TEPH01, TEPH02, M1 and M2 tephra layers show prevailing plane-parallel contacts and lack of internal grading. In CORE02, the occurrence of a level with erosional contacts between the ashy layers, the upward coarsening of the ash and the partial compaction of the levels indicate that this tephra layer includes flow deposits^17^.

The TEPH01 and TEPH02 ash in the CORE02 gravity core and the M1 and M2 ash of the Marsili1 core are characterized by a nearly equal amount of bubbles (16*–*13 area% in TEPH01 and M1 and 22–27 area% in TEPH02 and M2), as obtained from 2D measurements^18^ and by comparable matrix glass composition (Table S3). On the basis of (a) the similarity of these two different datasets and (b) the results of AMS^14^ Cdating of the sediments occurring at the base and top of the two tephra layers, previous authors concluded that the TEPH01-M1 and TEPH02-M2 tephra layers couples represent the proximal (CORE02) and distal (Marsili1) submarine outcrops of two distinct eruptive events occurred between 5 and 3 ka BP^18^. We named here these events MS1 and MS2, respectively. Most of the MS1 and MS2 clasts is characterized by poorly vesiculated, smoothed and fluidal scoriaceous ash with sub-circular bubbles and minor stretched clasts with tube-like, elongated vesicles^17^. Clasts with a clear evidence of magma-water interaction, e.g. hydration cracks and pitting, are rare^18^. The sources of the MS1 and MS2 eruptions are represented by three NNE-SSW aligned cones having summits around 670 m b.s.l. and located at a distance of about 700 and 1600 m from the proximal (CORE02) and distal (Marsili1) sites, respectively^17^ (Fig. 1c). This stratigraphic appraisal is unprecedented for deposits of deep submarine eruptions, and allows us to analyze the spatial variation of two tephra layers at different distance from the source(s) (Figs. 1c and 2).

## Results

The loose ash of TEPH01 (sample Mrs-21, the number refers to centimeters from the top of the core) and TEPH02 (samples Mrs-47, Mrs-62 and Mrs-73) and the two tephra layers M1 and M2 show the same dissolved water content (0.92 ± 7 *vs* 0.79 ± 9 and 0.92 ± 7 *vs* 0.79 wt.%; Table S2), as well as similar matrix glass compositions^18^ (Table S3). Loose ash from the CORE02 and Marsili1 cores has crystal contents of 39 area% (TEPH01) and 10 area% (M1) in MS1, and 13 area% (TEPH02) and 3 area% (M2) in MS2. Thus, the crystal amount of the ash in MS1 and MS2 decreases moving from CORE02 to Marsili1 (Figs. 1c and 2). The median grain-size (Mdφ) of MS1 is 0.63 in CORE02 and 3.44 in Marsil1, whereas MS2 has Mdφ of 0.67 in CORE02 and 2.54 in Marsil1 (Table S2 and Fig. 2). Then, a general decrease in grainsize occurs in MS1 and MS2 moving from CORE02 to Marsili1.

**Figure 3:**
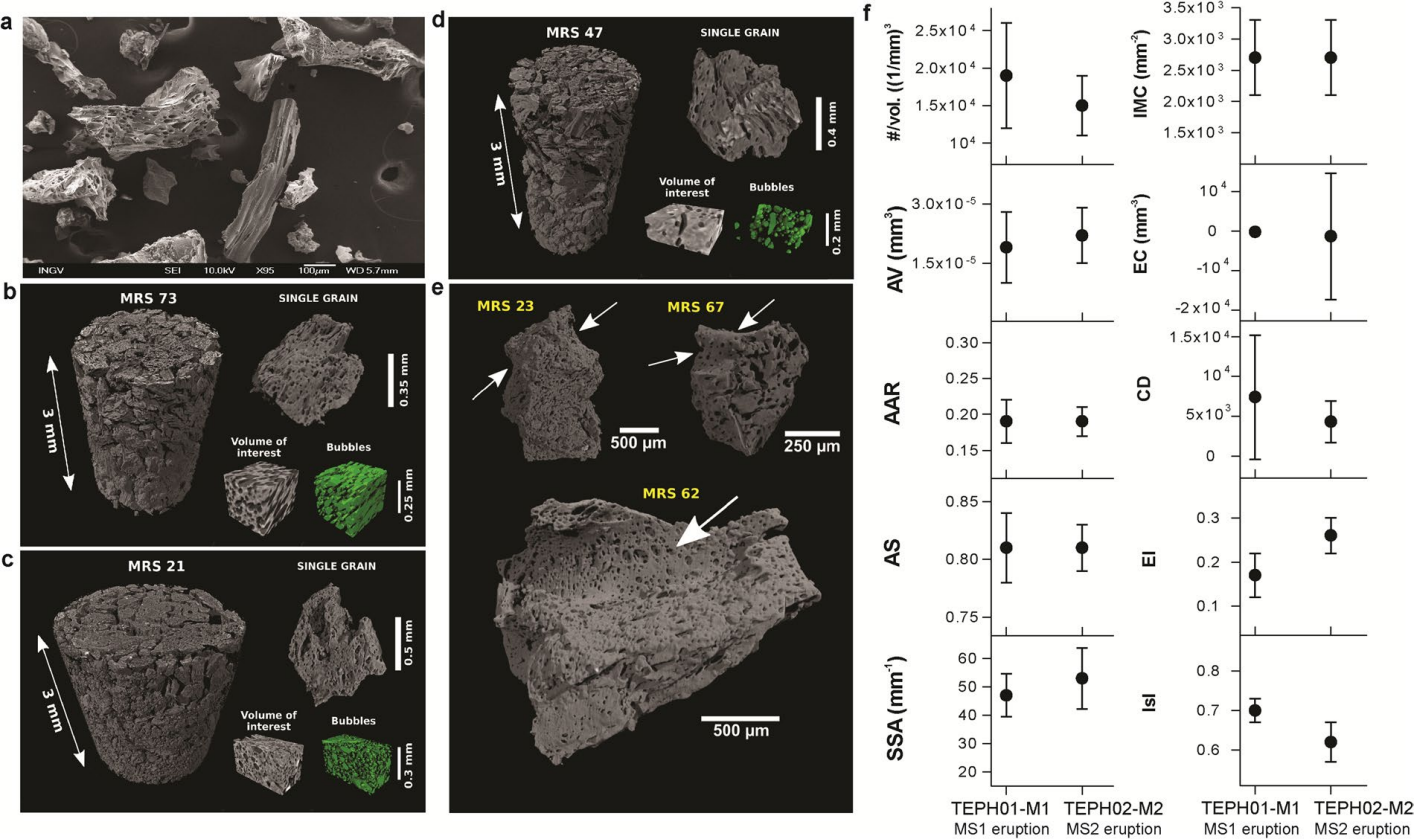
(**a**) SEM image of ash of MS1. (**b–d**) Volume renderings of selected samples from CORE02 proximal deposits with examples of a loose ash grain and extracted volume of interest; bubble phase segmented from the reported VOI is in green. (**e**) Single grains of ashes showing concave surfaces (arrows). Mrs-21 and -23 belong to the MS1 event, Mrs-47, -62, -67 and -73 to the MS2 eruption. (**f**) Average textural parameters of bubbles reported in Table S4 for the Marsili loose tephras: #/vol = bubbles per unit volume, AV = volume of bubbles, AAR = aspect ratio, AS = sphericity, SSA = specific surface area (SSA), IMC = integral of mean curvature, EC = Euler characteristic, CD = connectivity density, EI = elongation index, IsI = isotropy index.

**Figure 4:**
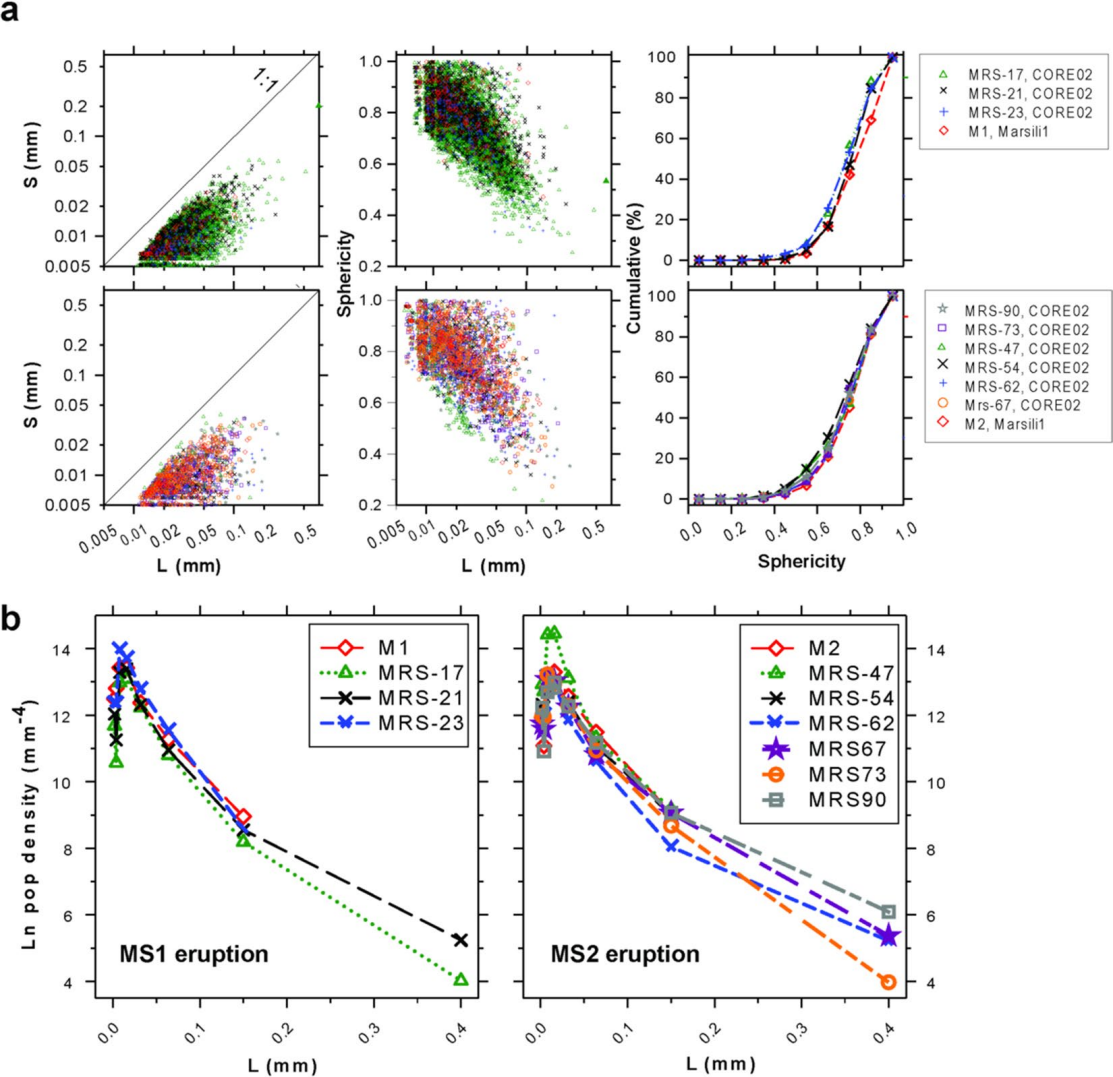
(**a**) Size and shape parameters of bubbles extracted from tomographic images for the MS tephra layers in both cores, considering only bubbles with the shortest size ≥5 µm. On the left column are reported the longest (L) *vs* shortest (S) segment in each bubble, in the middle, L *vs* Sphericity, and, on the right, the cumulative frequency distribution of Sphericity (ten classes). The size and shape of bubbles in the four loose tephra layers are very similar, since L and S have similar ranges and about 80–90% of bubbles have sphericity >0.5. These 3D data suggest similar processes of vesiculation. (**b**) Bubble size distributions (BSD) of MS1 (left) and MS2 (right), using maximum lengths in bubbles obtained from 3D image analysis.

Generally, the M1 and M2 ash particles are characterized by well-rounded to rectilinear boundaries with prevailing fluidal shapes (Fig. 3a). Rounded clasts are characterized by concave outward surfaces (Fig. 3e). The textural features of bubbles have been accurately quantified by 3D methods (see the Materials and methods section). Bubbles are generally isolated with only sporadic evidences of coalescence and show sub-circular to elongated shapes (Fig. 3a–d). The 3D amount of bubbles in all the analyzed samples of the four tephra layers is reported in Table S2. The bubble content is more commonly <35–40 vol.% (Fig. 2). Other 3D textural parameters measured for bubbles are reported in Table S4 and plotted in Fig. 3f. The MS1 and MS2 ashes show very similar 3D bubble textural parameters. These parameters include the number of bubbles per unit volume (#/vol.), average volume of bubbles (AV), average aspect ratio (AAR), average sphericity (AS), specific surface area (SSA), integral of mean curvature (IMC), elongation index (EI) and isotropy index (I). The only slight differences are evidenced by the Euler characteristic (EC) and connectivity density (CD) parameters (see Table S4 in Supplementary Materials). A further textural characterization of bubbles is provided by direct comparisons of their longest and shortest size and shape. These values are supported by the sphericity parameter calculated for the bubbles in the samples of the tephra layers at proximal and distal sites (Fig. 4a). 3D bubble size distributions are also very similar in MS1 and MS2 (Fig. 4b); they are characterized by a high number of bubbles with size <0.1 mm and a smaller amount with size between 0.1 and 0.4 mm. Most of the population density of bubbles with size >0.01 mm shows linear decreasing trends. Some samples show a break at size >0.1 mm, with trends characterized by a gentler decrease.

## Discussion

The similarities in the composition of the matrix glasses^18^, dissolved H_2_O content, and volume and 3D textural parameters of the bubbles demonstrate that the ash levels TEPH01 and TEPH02 (CORE02 core) and M1 and M2 (Marsili1 core) represent the proximal and distal deposits of two deep submarine explosive eruptions (MS1 and MS2). The 3D bubble parameters and the morphology of the MS1 and MS2 ash are also comparable, suggesting a similar eruptive mechanism. With respect to the two CORE02 tephra layers, which are characterized by thicker fall and volcanoclastic flow deposits^17^, the Marsili1 tephra layers are consistent with fall deposits^18^ (Figs. 1d and 2). We conclude that (a) fall deposits reduce their thickness moving away from the vent(s), as expected, and (b) volcanoclastic flow deposits emplace in proximal areas alone. The shape and amount (35–40 vol.%) of bubbles of the MS1 and of MS2 ash are similar to those observed in ash-sized pyroclasts from subaerial Strombolian and phreatomagmatic eruptions^21^ and significantly lower than that of subaerial and deep submarine silicic eruptions^8^. Subaerial Strombolian and phreatomagmatic eruptions have bubble content generally between 20 and 40 vol.% (e.g. Kilauea Iki, Miyakejima) with few examples of larger, up to 80 vol.%, contents (e.g., Stromboli paroxysms and Mauna Ulu lava fountains)^21^. Most of pyroclasts from subaerial plinian and deep submarine silicic eruptions has bubble content in the range 40–90 vol.%^21^. The bubble content of the MS1 and MS2 ash are consistent with the results of models on deep submarine, Strombolian-like eruption^2,13^. Since ash with evidence of magma-water fragmentation, i.e., ‘blocky’ clasts and glass shards, are poorly documented in the MS1 and MS2 deposits^17,18^, we propose that the studied Marsili eruptions occurred in a magmatic gas-rich environment with limited to locally absent interactions with seawater. This conclusion is also supported by the occurrence of ash particles with fluidal and concave-like boundaries, a feature consistent with the growth and successive fragmentation of larger bubbles during the eruption. These textures exclude a significant role of quenching of ash particles by granulation. Therefore, the quench of the MS1 and MS2 mainly developed within a magmatic gas and steam-rich zone and not in seawater, e.g. at the outer edge of the water-gas mixing zone^2^. The formation and raising of such gas-vapor expanding zone around the vent may displace away the seawater, as suggested by results of experimental studies^15^. Bubble size data in Fig. 4b suggest prevailing growth and coalescence processes, mainly of bubble with size >0.1 mm, and minor bubble nucleation or Ostwald ripening for sizes <0.01 mm. Also, the prevailing sub-spherical shape of the vesicles, the smoothed boundary of the ash, and the low amount of stretched vesicles indicate a bubble growth by decompressions at relatively low shear rate.

**Figure 5:**
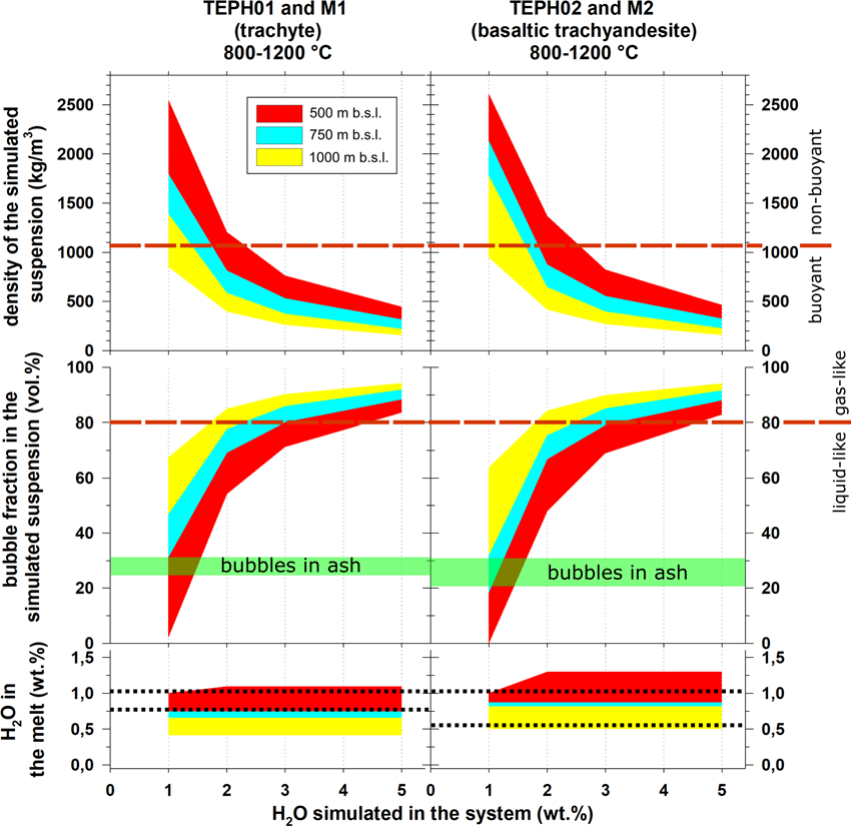
Bottom diagram: simulations of H_2_O solubility in the glass compositions of TEPH01 + M1 and TEPH02 + M2 tephra layers between 1200 and 800 °C for a vent at depth of 1000, 500 and 700–800 m b.s.l. considering a closed system with initial H_2_O contents of 1, 2, 3 and 5 wt.%; the range of dissolved H_2_O in the glass measured by FTIR is also reported by the dotted lines^17^. Middle diagram: simulations of bubble fraction assuming a closed suspension of crystals, melt and bubbles for TEPH01 and TEPH02 loose tephra layers between 1200 and 800 °C for a vent at depth of 1000, 500 and 700–800 m b.s.l.; the volume amount of bubbles (Table S1) measured by SR μCT is also reported; the 0.8 fraction of bubbles is the conservative threshold between a liquid- to gas-like behavior. Upper diagram: densities calculated from the previous numerical simulations accounting for glass compositions of MS1 and MS2 loose tephra layers between 1200 and 800 °C for a vent at depth of 1000, 500 and 700–800 m b.s.l.; suspensions with densities >1000 kg/m^3^ are buoyant in the seawater. Considering that the vent(s) of MS1 and MS2 is at 700–800 m b.s.l. (Fig. 1), the minimum amount of H_2_O required to erupt these magmas is close to 3 wt. %, either dissolved and degassed. Such H_2_O pre-eruptive content should discharge magmatic suspensions with a density of about 500 kg/m^3^.

The above reported conclusions are consistent with a ‘Strombolian’-like mechanism of magma ascent in the conduit^22–24^. The amount of bubbles of MS1 and MS2 ash represents only a fraction of that required to fragment the MS1 trachytic and MS2 basaltic trachyandesite magmas when magma-water interaction is limited or absent^2^. FTIR data did not detect significant amount of still dissolved CO_2_ (≤0.65 wt.%) in the glass of MS1 and MS2 ashes, whereas low amounts (≤ 1.50 wt.%) of H_2_O were measured in the same portions (Table S2). We computed the virtual amounts of H_2_O of 1, 2, 3 and 5 wt.% dissolved in two magmas with the same composition of the MS1 and MS2 glasses following a solubility model and by assuming a closed system at different depth (see Material and Methods). The results are shown in Fig. 5. At 500 and 1000 m b.s.l., the possible amount of dissolved H_2_O is around 0.7 wt.% and 1.2 wt.% for MS1 and MS2, respectively. At 700–800 m b.s.l., the maximum possible dissolved content of H_2_O is close to 1 wt.% (Fig. 5). Such estimates match the H_2_O content measured in the glass matrix of the analyzed ashes (Table S2) and indicate that the MS1 and MS2 magmas erupted in supersaturated conditions. Because elongated bubbles are subordinated in the MS1 and MS2 ash, a clear textural evidence of dominant strain-induced brittle-ductile fragmentation is lacking. We conclude that the MS1 and MS2 bubble break-up formed by viscous and capillary instabilities^25^. At 700–800 m b.s.l., the initial H_2_O content to attain a gas/magma ratio for fragmentation of 75/25^2^ should be between 2 and 3 wt.% (Fig. 5). Since the maximum amount of measured bubbles is lower than 35–40 vol.%, the MS1 and MS2 magmas were erupted with at least an additional ~45 vol.% of bubbles, a value unrecorded in the analyzed ash particles (Fig. 5). Therefore, the lacking fraction of bubbles should have a size equal or larger than that of the MS1 and MS2 ash particles. The occurrence of these hypothesized large bubbles is suggested by the external shape of the MS1 and MS2 ash particles, which show elliptical bubble shadows possibly representing larger, now lost bubbles (Fig. 3b-e). This interpretation agrees again with a Strombolian-like activity (Table S1) in which bubble-walls clasts are frequently observed and interpreted to result from magma squeezed among large gas bubbles^26,27^. According to the results from Fig. 5, a total amount of gas + dissolved 2–3 wt.% of H_2_O of the MS magmas allows a gas-pyroclasts mixture with a density around 500 kg/m^3^ to rise from a vent at 700–800 m b.s.l.. Magmas with a composition similar to that of MS1 and MS2 and more than 3 wt.% of dissolved and exsolved H_2_O may be erupted with even lower densities (Fig. 5). After the injection into the seawater, the cooling rate(s) and mixing between the volcanic plume and cold seawater determine the ability of the column to move upward^2–4,9^.

### Eruptive column and depositional mechanisms

**Figure 6:**
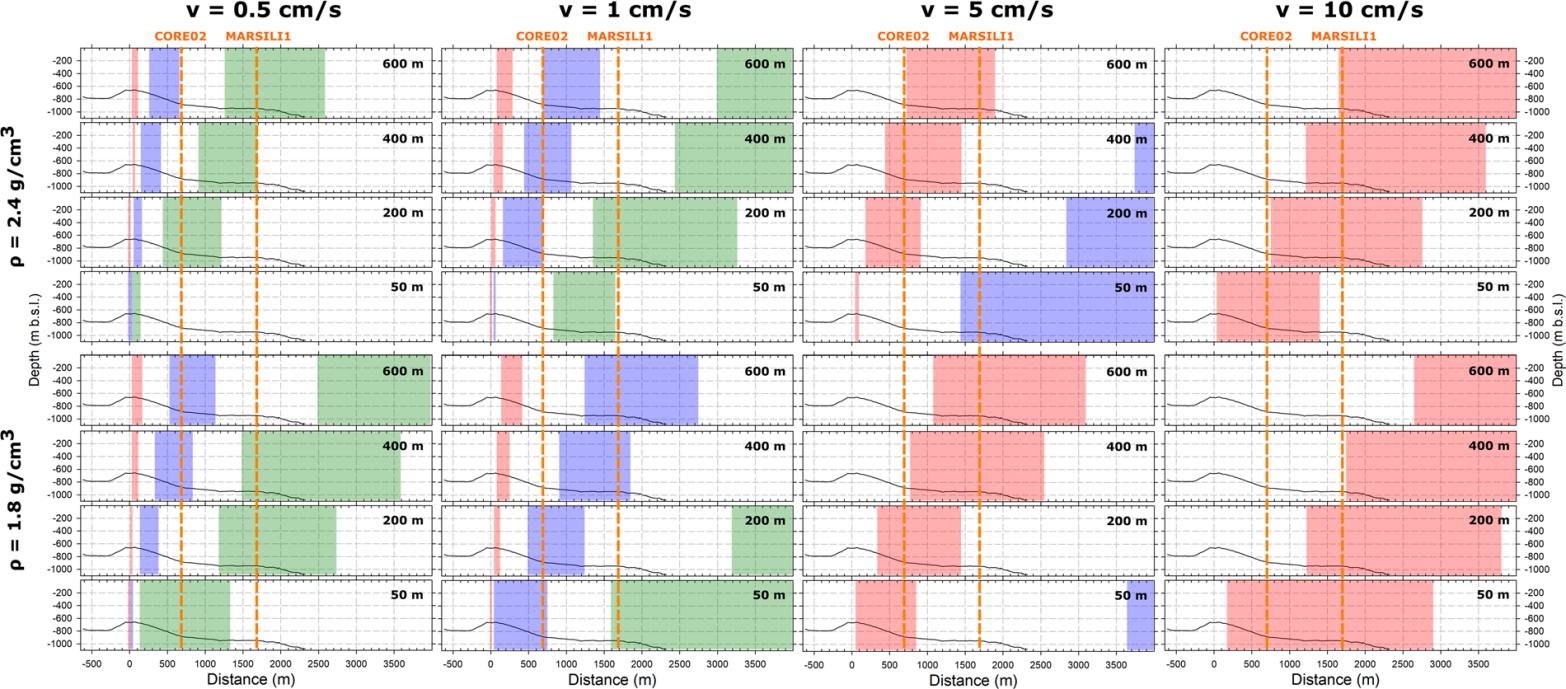
Simulations of settling distances as a function of clast size, shape and density. The morphological profile crosses the pyroclastic cones of Fig. 1d and the two gravity cores. Simulated clast sizes are 0.7 (light red), 0.2 (light blue) and 0.1 (light green) mm, while equant and tablet-like shapes correspond to the left and right vertical borders of each size. Simulated densities are 1800 and 2400 kg/m^3^, corresponding to relatively large and tiny clast sizes, respectively (Table S2). Simulated height of virtual volcanic plume above the vent are 50, 200, 400 and 600 m. This value, summed to the vertical differences between vent and the depth of the CORE02 and Marsili1 gravity cores corresponds to the total transport distance of ash. The computed time of sinking clasts have been used to calculate possible horizontal distances imposing four sea current velocities of 0.5, 1, 5 and 10 cm/s.

In the following, we estimate the height of the eruptive columns of the MS1 and MS2 eruptions. The height of submarine pyroclastic columns is poorly documented with the exception of that from Mata and NW-Rota 1, both showing meter-high columns^10,11^. At MS, the column height can be indirectly constrained by the sinking of MS1 and MS2 ash particles in seawater following a settling model (see Material and Methods). We simulate 0.5, 1, 5 and 10 cm/s lateral (horizontal) velocities, which account for the enlargement of the top of the column and the possible marine currents. The results of these computations are resumed in Fig. 6. MS1 column hosts loose clasts with sizes of about 0.65 mm in CORE02 and 0.1 mm in Marsili1 (Table S2). Assuming lateral velocities of 0.5 and 1 cm/s, particles with size of 0.7 mm and 0.1 mm, which are values representative of the TEPH01 clasts, are invariably extremely sluggish (Fig. 6). Conversely, clasts of 0.7 mm with equant to prismatic shapes may deposit in the site of CORE02 only if fallen from 200 and 400 m high plumes with a lateral velocity of 5 cm/s. Since relatively large particles are well represented by a density (*ρ*) of 1800 kg/m^3^, the most appropriate simulation is that consistent with a volcanic column of about 200 m. Tiny clasts (about 0.1 mm wide) like those of TEPH01 are well matched by two simulations: a) column height of about 50 m, *ρ* = 1800 kg/m^3^, and 1 cm/s of horizontal velocity, and b) column height of about 200 m, *ρ* = 2400 kg/m^3^ and 1 cm/s of horizontal velocity. Tiny clasts are more properly simulated by higher density; in turn, a plume height of 200 m represents the most plausible value (Fig. 6). As a result, the value of 200 m above the vent is a reasonable estimate of the column vertical expansion and this value is consistent with the depth and lateral distances of the MS1 deposits in the two gravity cores. This value of the column height is in the lower range of those estimated for subaerial Strombolian eruptions, which is between 100 m and <5 km^28^, and in the range of 50–400 m of the typical strombolian activity at Stromboli Island volcano^29^. A lateral velocity close to 5 cm/s for the larger ash particles in CORE02 can be explained from the vent proximity and, possibly, by the most significant effect of thermal enlargement of the plume. This effect is probably vanishing moving away from the vent for the tiny ashes of the Marsili1 core, where the sea currents prevail. Also, the finer ash particles found in may be related to the deposition from suspensions formed from pyroclastic density currents.

TEPH02 shows loose clasts with average size of 0.47, 0.67 and 0.9 mm in CORE02, and of 0.17 mm in Marsili1 (Fig. 2 and Table S2). Notably, clasts of 0.9 and 0.47 mm and *ρ* = 1800 kg/m^3^ are expected to fall from highest and lowest columns, respectively. Particles of about 0.2 mm, representing the tiny ash of TEPH02, are properly simulated by a column height of 400 to 600 m, *ρ* = 1800 kg/m^3^, and 1 cm/s of horizontal velocity (Fig. 6). Since tiny particles have larger density, the second simulation is the most reliable one. Simulation of 0.2 mm particles with density *ρ* = 2400 kg/m^3^ fits only a column of 50 m with a current of 5 cm/s. However, this simulation can be discarded because it explains only this particle size, being not consistent with the other ones. We conclude that the MS2 volcanic plume fluctuated in heights from about 200 to 400 m above the vent. This value is one to two orders of magnitude larger than that observed in video-recorded submarine eruptions, which is of few meters^12,30^. Therefore, the Marsili eruptions studied here were more energetic than those observed up to now in deep submarine environments.

A further important information from these Marsili tephra layers concerns the flow deposits recognized by previous studies^17^ in the MS1 and MS2 tephra layers of CORE02 (Fig. 2 and Table S2). In abyssal domains three main types of volcanoclastic flows can been distinguished: a) gravity and cold currents originated by remobilization of previously deposited tephras, b) gravity and cold currents from collapsing plume(s) during explosive eruption driven by the density contrast with seawater, and c) gravity and hot currents^2,13^. This latter occurrence corresponds to a pyroclastic flow similar to that emplaced in subaerial eruptions. The type a) flows has been documented several times (Table S1), whereas types b) and c) have been only hypothesized^2,9,13,31,32^, and the latter one have been reproduced only in laboratory with analog materials^15,33^. The proximal deposit of MS1 in CORE02 documents that before this eruption only a mud layer with a thickness of ~15 cm deposited in the MS area^17,18^ (Fig. 2). There are not direct stratigraphic contacts with a previously deposited or mobilized pyroclastic layer. The recognized semi-welded proximal layer in CORE02 consists exclusively of ash with a smaller grain-size with respect to the non-welded, loose tephra (Fig. 2 and Table S2). This proximal layer is free of sedimentary clasts^17^. This implies that the volcanic plume partially or entirely collapsed forming a density current that deposited directly on the pelagic sediments. This deposits account for the first 5–6 cm of TEPH01 (Table S2 and Fig. 2). Also, the lower and partly welded layer of TEPH01 has the largest amount of bubbles (Fig. 2 and Table S2). According to the conceptual model of deep submarine eruptions^2^, this Mrs-29 sample could represent the bubble (gas)-enriched and foam-like shallowest portion of the magma erupted at the beginning of the eruption. The very thick proximal deposit of MS2 in CORE02 represents a more complex picture. Although the length of the core prevents the inspection of its base layer(s), gently welded and loose ash clasts alternated for more than 50 cm (Fig. 2). These different levels also include erosive contacts and undulated surfaces^17^ (Table S2 and Fig. 2). All these observations indicate that the MS2 eruption was characterized by column collapses or, as alternative hypothesis, by collapses of lateral portions of the plume cooled more efficiently than the inner ones. We conclude that deep submarine eruptions can directly form density currents from their volcanic plumes, although their hot or cold nature remains a still open question.

Compared to the subaerial explosive eruptions, deep submarine events like those analyzed here are less able to raise up from the vent and less efficiently disperse their products in the surroundings areas; also, they can travel only few hundreds of meters as density flows^14^. In addition, gas-rich plumes of subaqueous eruption columns can entrain pyroclasts and raise upward^14^ so preventing an early magma-water interaction. Our results show that the products and eruptive mechanisms of the volcanic events recognized at MS may be not dissimilar to those recognized in subaerial events and, in particular, to those of Strombolian eruptions, but require a gas-rich cloud around the vent preventing the immediate quenching and granulation of the erupted pyroclasts by magma-water interaction.

## Materials and Methods

### Chemical and 2D textural characterizations

The bulk chemical composition of these tephra layers and their solid phases was measured using X-Ray Fluorescence (XRF), Instrumental Neutron Activation Analysis (INAA), back-scattered scanning electron microscopy (SEM-EDS) and electron microprobe analysis (EPMA-WDS)^17,18^. Here, we summarize only the salient characteristics. The dissolved amounts of H_2_O in matrix glasses of CORE02 were previously determined by Fourier Transform Infrared Spectroscopy (FTIR) analysis^17^; here, the same FTIR characterization has been performed on M-1 and M-2 tephra layers. 2D textural data have been already reported in^17,18^. Briefly, these two cores have been cut in two halves along their lengths and have been characterized by 2D analyses using mesoscopic surfaces and thin sections. 14 undisturbed tephra layers were considered, 8 from CORE02 and 6 from Marsili1 logs, respectively (Table S1 and Fig. 2). 2D quantitative analyses were performed on ashes embedded in epoxy resin, mounted on thin sections and then polished. Images were acquired using back-scattered scanning electron microscopy (BS-SEM), at variable magnifications^17,18^. Amount and numberof phases, i.e. bubble, glass and crystal, were measured by image analysis on BS-SEM micro-photographs^17,18,34–36^. All the salient 2D textural features and related chemical attributes of matrix glasses are summarized in Tables S2 and S3, respectively.

### Synchrotron X-ray computed microtomography (SR μCT) measurements

Three-dimensional (3D) analysis on 19 ashy samples, 13 from CORE02 and 6 from Marsili1 was performed by synchrotron X-ray microtomography at the SYRMEP beamline of the Elettra synchrotron facility in Basovizza (Trieste, Italy). The 3D characterization was performed by high-resolution SR μCT in phase-contrast mode^37^ using a polychromatic X-ray beam. Filters (1.5 mm Si + 1 mm Al) were applied to suppress the contribution of low energies in the beam spectrum. The detector consisted of a 16 bit, air-cooled, sCMOS camera (Hamamatsu C11440-22C) with a 2048 ×2048 pixel chip. Samples were mounted on the rotation stage without any specific preparation, setting a sample-to-detector distance of 150 mm. The effective pixel size of the detector was set at 1.98 μm × 1.98 μm, yielding a maximum field of view of about 4.0 mm × 4.0 mm. For each sample 1800 projections were acquired over a total scan angle of 180° and with an exposure time/projection of 2 s. Reconstruction of the 3D tomographic images was done by using the SYRMEP Tomo Project (STP) software suite, applying pre-reconstruction filters for reducing ring artefacts caused by detector inhomogeneities^38^. In order to improve the reliability of the segmentation process and further morphological analysis and to fully exploit the potential of phase-contrast SR μCT, a single distance phase-retrieval algorithm^39^ was applied to the projection images prior to reconstruction. Phase-retrieval, in combination with the Filtered Back-Projection algorithm^40^, allows to obtain the 3D distribution of the refraction index of samples with constant composition, and thus characterized by a constant ratio γ = δ/β between the real and imaginary parts of the refractive index at a given X-ray energy. It was demonstrated^41^ that this kind of algorithm can also be employed on multiphase volcanic rock samples imaged with a polychromatic synchrotron X-ray beam. In the case of our samples, best results for enhancing the contrast between bubbles and the bulk rock were obtained fixing γ = 100.

### Image processing and analysis

Segmentation and analysis of selected volumes of ash grains from the 19 samples were performed using the *Pore3D* software library^42^. Some examples are reported in Fig. 3. The tools available in *Pore3D* allowed a quantitative description of the morphology and topology of the grain components, as well as the analysis of the connectivity of the bubbles network by skeletonization approach^37,43^. Samples are made by ash grains with variable dimension, sometimes containing bubbles, sometimes bubble-free. From each of the 19 samples we selected from 2 to 10 grains containing bubbles. In order to perform the analysis, a volume of interest (VOI) was extracted from each grain, for a total of 140 VOIs with sizes variable from 55 × 62 ×35 to 407 × 460 × 295 voxels, corresponding to 0.0009 to 0.43 mm^3^. Since each selected VOI approximates the entire grain volume it can be considered representative of the grain heterogeneities and thus defined as Representative Elementary Volume (REV)^44^. After the extraction of the VOIs, data were filtered in order to remove noise and enhance edges by means of a 3D *Bilateral Filter*^42^, which smooths images and preserves edges applying a nonlinear combination of nearby image gray-level values. Image segmentation to obtain binary volumes containing only the objects of interest was performed using both the automatic MultiOtsuthresholding method^45^ on volumes containing more than 2 classes of object (e.g. bubbles, crystals and groundmass), and the simple Otsu method on volumes containing only groundmass and bubbles. The determined 3D amounts of bubbles in ashy samples are reported in Table S2. Density, specific surface area, integral of mean curvature and Euler characteristic (EC) of the bubbles were computed for all the investigated VOIs (Table S4). The EC parameter (expressed as mm^−3^) is an index of connectivity of a phase (bubble) network; typically, negative and positive values indicate connected and isolated phases, respectively. Analyses on each bubble were performed using the concept of maximal inscribed spheres^37^. We then calculated the number of bubbles, their volume, sphericity (ratio of the surface area of an equivalent sphere to the surface area of the object), aspect ratio (the ratio of the minimum and maximum axis of each bubble) and diameter of the maximal inscribed sphere. The preferred elongation and isotropy of the bubble networks were also evaluated on each VOI. The degree of bubbles connectivity was calculated using the *GVF* skeletonization algorithm^42^. After visual inspection, we applied a GVF scale value = 2 to up-sample the VOIs, in order to enrich the output skeleton and better fit the bubble network. The vesicle connectivity was investigated by determining the Connectivity Density (CD), a parameter derived from skeleton analysis. It is a scalar value representing the number of redundant connections normalized to the investigated volume. It is computed as (1 − (*n* − *b*))/*V*, where *n* is the number of pores and *b* the number of connections. Negative CD values indicate that the network is mainly constituted by isolated objects, whereas positive values indicate a high degree of connection. Volume visualization with volume rendering procedures was performed by means of the commercial software *VGStudio MAX 2.0* (Volume Graphics) to qualitatively characterize the samples.

## Dissolved and Exsolved H_2_O Models

The solubility of H_2_O in magmas was calculated considering temperature *T* of 1200, 1100, 1000, 900 and 800 °C and imposing *P* at 100 and 50 bars, two values representative of vents at 1000 and 500 m b.s.l., respectively. The H_2_O wt.% fractions dissolved and exsolved from the magmas were quantified using solubility models^46^. Then, the amount of exsolved H_2_O was calculated with respect to 1 m^3^ of magma. The moles of exsolved gas in a closed system were then used to calculate its volume *V* at imposed *T* and *P* conditions using the law of ideal gases. The ratios between gas and liquid magma were determined for variable *T*, *P* for 1, 2, 3 and 5 wt.% initial contents of H_2_O. The densities of the magmatic suspensions at high-*T* and -*P* were calculated assuming that all crystals (30–10 area% of plagioclase + pyroxene for MS1 and 18–10 area%, mainly plagioclase for MS2, see Table S2) were present before the eruption. These types and amount of minerals are unable to significantly shift the composition of the residual liquids with respect the corresponding bulk magmas^17,18^. On the whole, the two virtual suspensions at relevant magmatic *T* have *ρ* close to 2600 kg/m^3^. The densities of magma and exsolved gas, coupled with their relative amounts, allowed to estimate*ρ*. Noteworthy, possible outgassing inevitably requires an initial larger amount of H_2_O to reach the gas/magma ratio required for fragmentation. Hence, our model is conservative with respect to partial or full open systems.

## Sinking Model of Ash Particles

The sinking of ash particles was computed following the models proposed by^47^ applied on loose ashes. In the MS1 and MS2 deposits, the shape of loose clasts varies from nearly equant to prismatic^17,18^. We selected clast size of 0.7, 0.2 and 0.1 mm and considered different densities calculated using the DensityX program^48^ for the larger and bubble-richer clasts (1800 kg/m^3^) and the tiny relative bubble-poor particles (2400 kg/m^3^) (supplementary excel-spreadsheet). The depths of the supposed vent and of the CORE02 and Marsili1 gravity cores are at 670 m b.s.l., 839 m b.s.l. and 943 m b.s.l., respectively (Fig. 1c). Our simulations are for vents between 700 and 800 m depth, a range compatible with the depth of the Marsili vents taking into account the possible post-eruptive modifications^30^.

The minimum horizontal distance between the vent(s) and the two gravity cores is 700 m (CORE02) and 1600 m (Marsili1). The differences in depth of the vent and CORE02 and vent-Marsili1 are about 170 and 270 m, and the difference in depth between CORE02 and Marsili1 is about 100 m. We considered four different virtual heights of the volcanic plume above the vent (Fig. 1c), i.e. 50, 200, 400 and 600 m. These heights have been summed to 170 and 270 m to obtain the total vertical and horizontal distances. The computed timing of sinking from these heights have been used to calculate the horizontal distances imposing four different velocities of 0.5, 1, 5 and 10 cm/s.

## Supplementary information

Supplementary Information.

Supplementary Information2.

## Data Availability

All data are available in the Supplementary materials. The 5-m resolution DEM of the Marsili seamount of Fig. 1c is available on request to Guido Ventura (guido.ventura@ingv.it).
