# Supplementary material for: Deep sea explosive eruptions may be not so different from subaerial eruptions
Source: Sci Rep. 2020 Apr 21;10:6709. doi: 10.1038/s41598-020-63737-7 (PMC7174295; doi:10.1038/s41598-020-63737-7)
Supplement: Supplementary file 2 — Supplementary Information2. [file 41598_2020_63737_MOESM2_ESM.docx]

**Supplementary Materials for**

**Deep sea explosive eruptions may be not so different from subaerial eruptions**

Gianluca Iezzi^1,2^, Gabriele Lanzafame^3,4*^, Lucia Mancini^4^, Harald Behrens^5^, Stella Tamburrino^6^, Mattia Vallefuoco^6^, Salvatore Passaro^6^, Patrizio Signanini^1^, Guido Ventura^2^

^1^ Department of Engineering and Geology, G. D’Annunzio University, Chieti, Italy;

^2^ National Institute of Geophysics and Volcanology, Rome, Italy;

^3^ Department of Biological, Geological and Environmental Scieces, University of Catania, Italy.

^4^ Elettra-Sincrotrone Trieste S.C.p.A., Basovizza (Trieste), Italy;

^5^ Institute for Mineralogy, Leibniz University, Hannover, Germany;

^6^ Istitute of Marine Sciences (ISMAR-CNR), Napoli, Italy.

* corresponding author, email: [gabriele.lanzafame@gmail.com](mailto:gabriele.lanzafame@gmail.com)

The PDF file includes:

Table S1. Summary of explosive submarine eruptions at depths > 500 m b.s.l. from literature data.

Table S2. Tephra layers in the two cores and sampled levels as a function of their depth below the sea-floor, plus average grain-size parameters, amount of bubbles determined by both 2D and 3D (brackets) methods and amount of dissolved H2O and crystals.

Table S3. Average oxide compositions (wt. %) of glass matrix in tephras from the COR02 and Marsili1 logs.

Table S4. 3D textural features of bubbles.

**Table S1. Summary of explosive submarine eruptions at depths > 500 m b.s.l. from literature data.**

| Ocean | Geological  contest | Location | Volcano | Vent depth  (m bsl) | Sampling methods | Eruption style | Deposit(s) | Vesicles  (vol.%) | Composition | Reference |
| --- | --- | --- | --- | --- | --- | --- | --- | --- | --- | --- |
| Pacific  Ocean | intra-plate | Hawaii  USA | Lohi | 900 –  1200 | mainly dredged/  scooped  with ROV, plus 1 core | poseidic and  strombolian- to hawaian-like | lapilli- and ash-sized  blocky and bubble-wall loose pumiceous and scoriaceous clasts | 0 to 70 | basalt | 1-9 |
|  |  |  | North Arc  Ridge | 4000 –  4200 | dredged and  scooped  with ROV | strombolian-like | lapilli- and ash-sized  blocky and bubble-wall loose scoriaceous clasts  potential  density currents | 0 to 70 | nephelinite, basanite  and  basalt | 10 |
|  | convergent  margin | Marianna arc  (East of  Philippines) | NW-Rota1 | 530 -  560  (filmed) | dredged and  scooped  with ROV | strombolian-like  (10^0^ to 10^1^  m columns) | bomb-, lapilli- and ash-sized loose clasts  density currents | 12-38 | basaltic  andesite | 11-16 |
|  |  | Lau basin,  Tonga trench  (South of  Samoa) | West Mata | 1200 -  2200  (filmed) | scooped  with ROV,  but un-analyzed | strombolian- to to hawaian-like  (10^0^ to 10^1^  m columns) | spatter, lapilli  and ashes from fallout  density currents | undetermined | boninite | 17-20 |
|  |  | Juan de  Fuca ridge  (West of Washington) | Axial  Seamount | 1400 -  2500 | push cores of sedimentary and volcanic layers | strombolian-like  and phreatomagmatic | bubble-wall  loose lapilli clasts,  dispersed up to 5 km | 5 - 50 | basalt | 21-24 |
|  |  | Izu-Bonin arc  (South of  Japan) | Sumisu Volcanic Complex, domes B and C | 870 -  1300 | dredged  with ROV | induced by dome disruption  (at depths  > 500 m) | giant (> 1m) and loose pumiceous clasts | 4 - 77 | rhyolite | 25 |
|  |  | Kermadec arc  (North of  New Zeland) | Healy | 550 -  1700 | dredged | large explosive eruption (some km^3^) | breccia- and lapilli-sized pumicrous loose clasts | 35 - 95 | rhyodacite | 26-28 |
|  |  |  | Raoul SW | 500 -  1200 | dredged (only clasts 16 – 32 mm) | large explosive eruption (some km^3^) | loose pumiceous clasts | 63-92 | rhyolite | 26, 29 |
|  |  |  | Macauley | ~ 1100 | dredged | tangaroan | loose pumiceous clasts  possible density currents | 10-95 | rhyodacite | 28, 30 |
|  |  |  | Havre | 640 -  1400 | dredged and  push cores | effusive-related paroxysms | giant and loose pumiceous clasts (> 1 m); block-, lapilli- and ash-sized loose deposits  possible (vertical) density currents | 65-90 | rhyolite | 26, 29, 31 |
|  | divergent  margin | West of Northern California Oregon Margin | Gorda  ridge | > 2800 | dredged and  push cores | strombolian-  like | lapilli- and ash-sized  blocky and bubble-wall loose clasts | ~ 15 | basalt | 32 |
| Atlantic  Ocean | divergent  margin | Mid-Atlantic  Ridge  (West and  South of  Azores) | Mid-Atlantic Ridge | 1500 - 1900 | dredged/  scooped  with ROV | unspecified | lapilli- and ash-sized  blocky and bubble-wall loose clasts and hyaloclastites | 35-50 | basalt | 33 |
|  |  |  | West of  Azores | 500 - 1700 | dredged/  scooped  with ROV | unspecified | lapilli- and ash-sized  scoriaceous and pumiceous (loose?) clasts | 30 - 80 | basalt | 34 |
|  |  |  | South of the Azores | 550 - 1750 | dredged | unspecified magmatic explosive activity, plus hydrovolcanic | lapilli- and ash-sized  scoriaceous and bubble-wall loose scoriaceous clasts  possible density currents | 27-65% | basalt | 35 |
| Arctic  Ocean | divergent  margin | Arctic basin  (north of Svalbard) | ACAVE | > 3400 | dredged/suctioned  with ROV | strombolian-like | lapilli- and ash-sized  blocky and bubble-wall loose clasts | ≤ 5 | basalt | 36-38 |
| Mediterranean sea | early divergent, then convergent  margin | Tyrrhenian Sea  (Eest of  Italy) | Marsili  volcano | 700/800 | gravity  cores | strombolian-like | ash-sized  pumiceous loose and gently welded clasts  density currents | 5 - 24 | basaltic trachy-andesite to trachyte | 39-40 |

**References for table S1**

1 Clague, D. A. Accumulation rates of volcaniclastic deposits on Loihi Seamount. *Bull. Volcan.***71**, 705–710 (2009).

2 Clague, D. A., Davis, A. S., Bischoff, J. L., Dixon, J. E. & Geyer, R. Lava bubble-wall fragments formed by submarine hydrovolcanic explosions on Loihi Seamount and Kilauea Volcano. *Bull. Volcan.***61**, 473-449 (2000).

3 Barreyre T., Soule, S. A. &Sohn, R. A. Dispersal of volcaniclasts during deepsea eruptions: Settling velocities and entrainment in buoyant seawater plumes. *J. Volcan. Geotherm. Res.***205**, 84–93 (2011)

4 Clague, D. A., Batiza, R., Head, J. W. I. & Davis, A.S.. Pyroclastic and hydroclastic deposits on Loihi Seamount, Hawaii. *Explosive Subaqueous Volcanism (eds*White, J. D. L., Smellie, J. L. &Clague, D. A.) Am. Geophys. Union, Geophys. Monograph Series, 73–95 (2003).

5 Schipper, C. I. & White, J. D. L No depth limit to hydrovolcaniclimu o Pele: analysis of limu from Loihi Seamount, Hawaii. *Bull. Volcan.***72**, 149–164 (2010).

6 Schipper, C.I., White, J.D.L. & Houghton, B.F. Syn- and post-fragmentation textures in submarine pyroclasts from Loihi Seamount, Hawaii. *J. Volcan. Geotherm. Res.*191, 93–106 (2010).

7 Schipper, C. I., White, J. D. L., Houghton, B. F., Shimizu, N. & Stewart, R. B. Esplosive submarine eruptions driven by volatile-coupled degassing at Lō`ihi Seamount, Hawaii. *Earth Plan. Sci. Lett.* (2010) **295**, 497–510.

8 Schipper, C. I., White, J. D. L., Houghton, B.F., Shimizu, N. & Stewart, R.B., 2010c. “Poseidic” explosive eruptions at Loihi Seamount, Hawaii. *Geology***38**, 291–294 (2010).

9 Schipper C. I., White J. D. L., Houghton B.F. Textural, geochemical, and volatile evidence for a Strombolian-like eruption sequence at Lō`ihi Seamount, Hawai`i. *J. of Volcan. Geotherm. Res.***207**, 16-32 (2011).

10 Davis, A. S. &Clague, D. A. Volcaniclastic deposits from the North Arch volcanic field, Hawaii: explosive fragmentation of alkalic lava at abyssal depths. *Bull. Volcan.***68**, 294–307 (2006).

11 Chadwick, W.W. et al. Direct video and hydrophone observations of submarine explosive eruptions at NW Rota-1 volcano, Mariana arc. *J. Geophys. Res.***113**, B08S10 (2008).

12 Deardorff, N. D., Cashman, K. V. & Chadwick, W. W. Observations of eruptive plume dynamics and pyroclastic deposits from submarine explosive eruptions at NW Rota-1, Mariana arc. *J. Volcan. Geotherm. Res.***202**, 47-59 (2011).

13 Schnur, S. R., et al. A decade of volcanic construction and destruction at the summit of NW Rota-1 seamount: 2004–2014. *J. Geophys. Res. Solid Earth* **122**, 1558–1584 (2017).

14 Embley, R. W. et al. Long-term eruptive activity at a submarine arc volcano. *Nature***441**, 494–497 (2006).

15 Chadwick, W. W., Dziak, R.P., Haxel, J.H., Embley, R.W. & Matsumoto, H. Submarine landslide triggered by volcanic eruption recorded by in-situ hydrophone. *Geology***40**, 51–54 (2012).

16 Walker, S.L. et al. Eruption-fed particle plumes and volcaniclastic deposits at a submarine volcano: NW-Rota-1, Mariana Arc: J. of Geophys. Res. **113**, B08S11 (2008).

17 Resing J. A. et al. Active submarine eruption of boninite in the northeastern Lau Basin. *Nature Geosci.***4**, 799–806(2011).

18 Clague, D. A. et al. Volcanic morphology of West Mata Volcano, NE Lau Basin, based on high resolution bathymetry and depth changes. *Geochem. Geophys. Geosyst.***12**, QOAF03 (2011).

19 Embley, R.W., et al. Eruptive modes and hiatus of volcanism at West Mata seamount, NE Lau basin: 1996–2012. *Geochem. Geophys. Geosyst.***15**, 4093–4115 (2014).

20 Dziak, R. P. et al. Long-term explosive degassing and debris flow activity at West Mata submarine volcano. *Geophys. Res. Lett.***42**, 1480–1487 (2015).

21 Clague, D. A., Paduan, J. B. & Davis, A. S. Widespread Strombolian eruptions of mid-ocean ridge basalt. *J. Volcan. Geotherm. Res.***180**, 171–188 (2009).

22 Helo, C., Longpre´, M. A., Shimizu, N., Clague, D.A. & Stix, J. Explosive eruptions at mid-ocean ridges driven by CO_2_-rich magmas. *Nature Geosci.***4**, 260-263 (2011).

23 Clague, D. A. et al. Geologic history of the summit of Axial Seamount, Juan de Fuca Ridge, *Geochem. Geophys. Geosyst.***14**, 4403–4443 (2013).

24 Portner R. A, Clague D. A., Helo C., Dreyer B. M. &Paduan J. B. Contrasting styles of deep-marine pyroclastic eruptions revealed from Axial Seamount push core records. *Earth Plan. Sci. Lett.***423**, 219–231 (2015).

25 Allen S. R., Fiske R. S. & Tamura Y. Effects of water depth on pumice formation in submarine domes at Sumisu, Izu-Bonin Arc, western Pacific. *Geology***38**, 391–394 (2010).

26 Rotella M. D., Wilson C. J. N., Barker S. J., Schipper C.I., Wright I.C., Wysoczanski R. J. Dynamics of deep submarine silicic explosive eruptions in the Kermadec arc, as reflected in pumice vesicularity textures. *J. Volcan. Geotherm. Res.* **301**, 314-332 (2015).

27 Wright, I. C., Gamble J. A., Shane P. A. R. Submarine silicic volcanism of the Healy caldera, southern Kermadecarc (SW Pacific): I – volcanology and eruption mechanisms. *Bull. Volcanol.***65**, 15–29 (2003).

28 Barker, S. J., Rotella, M. D.,Wilson, C. J. N., Wright, I. C.,Wysoczanski, R. J. Contrasting pyroclast density spectra from subaerial and submarine silicic eruptions in the Kermadec arc: implications for eruption processes and dredge sampling. *Bull. Volcanol.***74**, 1425-1443 (2012).

29 Carey R. et al. The largest deep-ocean silicic volcanic eruption of the past century. Science Adv. **4**, e1701121 (2018).

30 Rotella M. D., Wilson C. J. N., Barker S. J. & Wright I.C. Highly vesicular pumice generated by buoyant detachment of magma in subaqueous volcanism. *Nature Geosci.***6**, 129–132 (2013).

31 Murch, A. P., White, J. D. L. & Carey, R. J. Characteristics and Deposit Stratigraphy of Submarine-Erupted Silicic Ash, Havre Volcano, Kermadec Arc, New Zealand. *Front. Earth Sci.***7**, 1-21 (2019).

32 Clague, D. A., Batiza, R., Head, J. W. I. & Davis, A.S.. Pyroclastic and hydroclastic deposits on Loihi Seamount, Hawaii. *Explosive Subaqueous Volcanism (eds*White, J. D. L., Smellie, J. L. &Clague, D. A.) Am. Geophys. Union, Geophys. Monograph Series, 73–95 (2003).

33 Hekinian, R. et al. Deep sea explosive activity on the Mid-Atlantic Ridge near 34 degrees 50′N; magma composition, vesicularity and volatile content. *J. Volcanol. Geotherm. Res.***98**, 49–77 (2000).

34 Fouquet, Y. et al. Extensive volcaniclastic deposits at the Mid-Atlantic Ridge axis; results of deepwater basaltic explosive volcanic activity? *Terra Nova***10**, 280–286 (1998).

35 Eissen, J. P., Fouquet, Y., Hardy, D. &Ondreas, H. Recent MORB volcaniclastic explosive deposits formed between 500 and 1700 m.b.s.l. on the axis of the Mid-Atlantic Ridge, South of the Azores. In: Explosive Subaqueous Volcanism (eds White J. D. L., Smellie, J. L., Clague D. A.) Am. Geophys. Union, Geophys. Monograph Series **140**, 143–166 (2003).

36 Ruth, D.C.S. & Calder, E.S. Plate tephra: Preserved bubble walls from large slug bursts during violent Strombolian eruptions. *Geology***42** (1): 11–14 (2014), [doi:10.1130/G34859.1](https://doi.org/10.1130/G34859.1).

37 Sohn, R.A. et al. Explosive volcanism on the ultraslow-spreading Gakkel ridge, Arctic Ocean. *Nature***453**, 1236-1238 (2008).

38 Pontbriand, C.W. et al. Effusive and explosive volcanism on the ultraslow-spreading Gakkel Ridge, 85° E. *Geochem. Geophys. Geosyst.* **13**,Q10005 (2012).

39 Iezzi G. et al. First documented deep submarine explosive eruptions at the Marsili Seamount (Tyrrhenian Sea, Italy): A case of historical volcanism in the Mediterranean Sea. *Gondwana Res.***25**, 764–774 (2014).

40 Tamburrino S. et al. The proximal marine record of the Marsili Seamount in the last 7ka (Southern Tyrrhenian Sea, Italy): Implications for the active processes in the Tyrrhenian Sea back-arc. *Glob. Plan. Change***133,** 2–16 (2015).

**Table 2S. Tephra layers in the two cores and sampled levels as a function of their depth below the sea-floor, plus average grain-size parameters, amount of bubbles**

| log | CORE02 | | | | | | | | | Marsili1 | | | | | | | |
| --- | --- | --- | --- | --- | --- | --- | --- | --- | --- | --- | --- | --- | --- | --- | --- | --- | --- |
| tephra | sample  label | depth  (cm) | deposit | Mdφ | σφ | bubbles  area%  **vol.%** | dissolved  H_2_O  (wt.%) | dissolved  CO_2_  (wt.%) | crystals  area% | sample  label | depth bsf  (cm) | deposit | Mdφ | σφ | bubbles  area%  **vol.%** | dissolved  H_2_O  (wt.%) | crystals  area% |
| TEPH01  ~ 3 ka | Mrs-17 | 17 | loose |  |  | **32(9)** |  |  |  | M1 | 18 | loose | 3.44 | 1.86 | 13(11)  **27(14)** | 0.79(0.09) | 10(14) |
|  | Mrs-21 | 21 | loose | 0.63 | 0.97 | 16  **26(7)** | 0.97 | 0.659 | 39 |  |  |  |  |  |  |  |  |
|  | Mrs-23 | 23 | loose |  |  | **25(6)** |  |  |  |  |  |  |  |  |  |  |  |
|  | Mrs-27 | 27 | welded | 1.72 | 1.21 | 13  **29(3)** | 0.87 | 0.114 | 43 |  |  |  |  |  |  |  |  |
|  | Mrs-29 | 29 | welded |  |  | **49(19)** |  |  |  |  |  |  |  |  |  |  |  |
| TEPH02  ~ 5 ka | Mrs-41 | 41 | welded | 2.22 | 1.30 | 5  **14(7)** | 0.78 |  | 16 | M2 | 26 | loose | 2.54 | 1.24 | 20(14)  **22(7)** | 0.73(0.15) | 3(4) |
|  | Mrs-47 | 47 | loose | 0.56 | 0.77 | 20  **25(10)** | 0.95 |  | 10 |  |  |  |  |  |  |  |  |
|  | Mrs-54 | 54 | loose |  |  | **27(10)** |  |  |  |  |  |  |  |  |  |  |  |
|  | Mrs-62 | 62 | loose | 1.29 | 1.09 | 13  **34(4)** | 0.57 | 0.407 | 18 |  |  |  |  |  |  |  |  |
|  | Mrs-67 | 67 | loose |  |  | **31(7)** |  |  |  |  |  |  |  |  |  |  |  |
|  | Mrs-73 | 73 | loose | 0.17 | 0.81 | 24  **32(6)** | 0.5 |  | 11 |  |  |  |  |  |  |  |  |
|  | Mrs-82 | 82 | welded | 1.88 | 1.37 | 8  **16(6)** | 0.53 |  | 13 |  |  |  |  |  |  |  |  |
|  | Mrs-90 | 90 | loose |  |  | **27(10)** |  |  |  |  |  |  |  |  |  |  |  |
|  | Mrs-95 | 95 | welded | 3.04 | 1.76 | 7  **18(5)** | 0.95 |  | 14 |  |  |  |  |  |  |  |  |

Footnotes: stratigraphic description of cores, physical state of deposit, 2D average grain-size (Mdφ) and related (σφ) dispersion, 2D amount of bubbles, volatile contents dissolved in glasses of CORE02 and amount of crystals are from^17,18^. The content of volatiles in the two shallowest Marsili1 tephras are measured in this study.

**Table S3. Average oxide compositions (wt. %) of glass matrix in tephras from the COR02 and Marsili1 logs.**

|  | | | | | | |
| --- | --- | --- | --- | --- | --- | --- |
| tephra | TEPH01 | | TEPH02 | | TEPH03 | TEPH04 |
| log | CORE02 | Marsili1 | CORE02 | Marsili1 | Marsili1 | Marsili1 |
| sample  label | mean  (2 tephras) | M1 | mean  (6 tephras) | M2 | M3 | mean  (6 tephras) |
| SiO_2_ | 62.74 (0.68) | 64.45 (1.51) | 55.97 (0.66) | 55.77 (0.6) | 53.88 (0.82) | 54.11 (0.72) |
| TiO_2_ | 1.01 (0.03) | 0.91 (0.21) | 1.29 (0.03) | 1.34 (0.18) | 1.37 (0.16) | 1.17 (0.05) |
| Al_2_O_3_ | 16.36 (0.08) | 17.25 (1.94) | 16.16 (0.31) | 16.56 (0.21) | 16.02 (0.31) | 16.21 (0.11) |
| FeO | 4.55 (0.23) | 4.08 (0.92) | 7.47 (0.29) | 8.07 (0.47) | 8.63 (0.43) | 8.35 (0.21) |
| MnO | 0.12 (0.01) | 0.14 (0.12) | 0.15 (0.01) | 0.18 (0.13) | 0.14 (0.15) | 0.24 (0.05) |
| MgO | 1.63 (0.14) | 0.99 (0.32) | 3.72 (0.13) | 3.74 (0.2) | 4.68 (0.48) | 4.51 (0.41) |
| CaO | 3.61 (0.16) | 3.62 (1) | 7.04 (0.24) | 7.65 (0.22) | 9.20 (0.71) | 9.08 (0.69) |
| Na_2_O | 4.72 (0.1) | 4.41 (0.71) | 4.15 (0.09) | 3.99 (0.24) | 3.44 (0.29) | 3.37 (0.11) |
| K_2_O | 3.44 (0.09) | 3.48 (0.75) | 2.00 (0.09) | 1.90 (0.08) | 2.01 (0.2) | 2.28 (0.29) |
| P_2_O_5_ | 0.27 (0.01) | 0.25 (0.15) | 0.50 (0.02) | 0.49 (0.2) | 0.40 (0.25) | 0.36 (0.08) |
| Cl | 0.28 | 0.33 (0.14) | 0.17 (0.01) | 0.20 (0.04) | 0.18 (0.05) | 0.18 (0.06) |
| total | 98.71 (0.17) | 97.72 (2.07) | 98.62 (0.85) | 98.45 (1.27) | 98.78 (1.56) | 97.63 (0.98) |
| H_2_O | 0.92 | 0.79 | 0.65 | 0.73 | - | - |
| alkalies | 8.16 (0.07) | 7.93 (0.38) | 6.14 (0.16) | 5.91 (0.28) | 5.46 (0.39) | 5.67 (0.36) |
| TAS | Tr | Tr | BTrA - TrA | TrA | BTrA | BTrA |

Footnotes: Tr = trachyte; BTrA = basaltic trachy-andesite; TrA = trachy-andesite. Chemical data are from^17,18^

**Table S4. 3D textural features of bubbles**.

| tephra | sample  label | deposit | #/vol.  (1/mm^3^) | AV  (mm^3^) | AAR | AS | SSA  (mm^-1^) | IMC  (mm^-2^) | EC  (mm^-3^) | CD | EI | IsI |
| --- | --- | --- | --- | --- | --- | --- | --- | --- | --- | --- | --- | --- |
| TEPH01 | MRS-17 | loose | 1.1*10^4^ | 2.8*10^-5^ | 0.21 | 0.77 | 47.6 | 2.7*10^3^ | 4.2*10^3^ | 4.6*10^3^ | 0.12 | 0.74 |
|  | MRS-21 | loose | 1.6*10^4^ | 1.8*10^-5^ | 0.21 | 0.80 | 36.6 | 2.2*10^3^ | 1.2*10^4^ | 2.8*10^3^ | 0.18 | 0.68 |
|  | MRS-23 | loose | 2.8*10^4^ | 7.5*10^-6^ | 0.15 | 0.81 | 48.7 | 3.5*10^3^ | 1.4*10^4^ | 3.1*10^3^ | 0.23 | 0.68 |
|  | MRS27 | welded | 8.7*10^4^ | 3.5*10^-5^ | 0.22 | 0.79 | 46.9 | 2.1*10^3^ | 2.7*10^3^ | 3.1*10^3^ | 0.17 | 0.72 |
|  | MRS29 | welded | 4.3*10^4^ | 1.7*10^-4^ | 0.13 | 0.82 | 65.5 | 1.6*10^3^ | -2.6*10^4^ | 2.7*10^4^ | 0.09 | 0.83 |
|  | M-1 | loose | 1.9*10^4^ | 2.2*10^-5^ | 0.17 | 0.84 | 54.9 | 2.5*10^3^ | -7.7*10^3^ | 1.9*10^4^ | 0.15 | 0.70 |
|  | **average loose**  **(s.d.)** | | **1.9*10^4^**  **(0.7*10^4^)** | **1.9*10^-5^**  **(0.9*10^-5^)** | **0.19**  **(0.03)** | **0.81**  **(0.03)** | **47.0**  **(7.6)** | **2.7*10^3^**  **(0.6*10^3^)** | **-2.0*10^2^**  **(4.0*10^2^)** | **7.4*10^3^**  **(7.8*10^3^)** | **0.17**  **(0.05)** | **0.70**  **(0.03)** |
|  | **average all**  **(s.d.)** | | **3.4*10^4^**  **(2.8*10^4^)** | **4.7*10^-5^**  **(6.1*10^-5^)** | **0.18**  **(0.04)** | **0.81**  **(0.02)** | **50.0**  **(9.6)** | **2.4*10^3^**  **(0.6*10^3^)** | **-5.6*10^3^**  **(1.0*10^4^)** | **9.9*10^3^**  **(10.0*10^3^)** | **0.16**  **(0.05)** | **0.73**  **(0.06)** |
| TEPH02 | MRS41 | welded | 1.9*10^4^ | 7.5*10^-6^ | 0.24 | 0.84 | 26.5 | 1.9*10^3^ | 1.3*10^4^ | 1.7*10^3^ | 0.15 | 0.74 |
|  | MRS47 | loose | 2.4*10^4^ | 1.2*10^-5^ | 0.19 | 0.83 | 73.0 | 4.0*10^3^ | -3.8*10^4^ | 2.3*10^3^ | 0.21 | 0.69 |
|  | MRS-54 | loose | 1.4*10^4^ | 1.7*10^-5^ | 0.18 | 0.80 | 49.1 | 2.6*10^3^ | 5.9*10^3^ | 3.0*10^3^ | 0.30 | 0.57 |
|  | MRS-62 | loose | 1.1*10^4^ | 3.2*10^-5^ | 0.15 | 0.79 | 61.2 | 2.9*10^3^ | -6.2*10^2^ | 9.0*10^3^ | 0.28 | 0.62 |
|  | MRS-67 | loose | 1.4*10^4^ | 2.6*10^-5^ | 0.17 | 0.79 | 51.9 | 2.5*10^3^ | 4.7*10^3^ | 6.5*10^3^ | 0.28 | 0.60 |
|  | MRS-73 | loose | 1.3*10^4^ | 2.7*10^-5^ | 0.19 | 0.79 | 55.7 | 2.7*10^3^ | 5.1*10^3^ | 4.6*10^3^ | 0.31 | 0.57 |
|  | MRS-82 | welded | 1.8*10^4^ | 9.6*10^-6^ | 0.23 | 0.82 | 31.6 | 2.0*10^3^ | 9.0*10^3^ | 8.4*10^2^ | 0.22 | 0.66 |
|  | MRS-90 | loose | 1.3*10^4^ | 2.3*10^-5^ | 0.21 | 0.81 | 44.3 | 2.1*10^3^ | 5.2*10^3^ | 2.4*10^3^ | 0.23 | 0.61 |
|  | MRS-95 | welded | 1.9*10^4^ | 9.5*10^-6^ | 0.21 | 0.84 | 36.6 | 2.4*10^3^ | 1.1*10^4^ | 1.4*10^3^ | 0.18 | 0.72 |
|  | M-2 | loose | 1.8*10^4^ | 1.7*10^-5^ | 0.21 | 0.85 | 41.3 | 2.4*10^3^ | 8.4*10^3^ | 2.5*10^3^ | 0.21 | 0.67 |
|  | **average loose**  **(s.d.)** | | **1.5*10^4^**  **(0.4*10^4^)** | **2.2*10^-5^**  **(0.7*10^-5^)** | **0.19**  **(0.02)** | **0.81**  **(0.02)** | **53.8**  **(10.8)** | **2.7*10^3^**  **(0.6*10^3^)** | **-1.3*10^3^**  **(1.6*10^4^)** | **4.3*10^3^**  **(2.6*10^3^)** | **0.26**  **(0.04)** | **0.62**  **(0.05)** |
|  | **average all**  **(s.d.)** | | **1.6*10^4^**  **(0.4*10^4^)** | **1.8*10^-5^**  **(0.8*10^-5^)** | **0.20**  **(0.03)** | **0.82**  **(0.02)** | **47**  **(14.1)** | **2.6*10^3^**  **(0.6*10^3^)** | **-2.4*10^1^**  **(2.1*10^2^)** | **4.2*10^3^**  **(2.8*10^3^)** | **0.24**  **(0.05)** | **0.65**  **(0.06)** |
| TEPH03 | M-3 | loose | 1.1*10^4^ | 1.3*10^-5^ | 0.26 | 0.82 | 23.1 | 1.4*10^3^ | 8.8*10^3^ | 1.3*10^3^ | 0.15 | 0.71 |
| TEPH04 | M4-top | loose | 1.1*10^4^ | 9.3*10^-6^ | 0.26 | 0.86 | 17.9 | 1.4*10^3^ | 1.1*10^4^ | 7.7*10^1^ | 0.12 | 0.78 |
|  | M4-bottom  bis | loose | 1.1*10^4^ | 2.8*10^-5^ | 0.20 | 0.79 | 21.3 | 1.3*10^3^ | 7.0*10^3^ | 5.4*10^2^ | 0.19 | 0.67 |
|  | **average**  **(s.d.)** | | **1.1*10^4^** | **1.8*10^-5^**  **(1.3*10^-5^)** | **0.23**  **(0.04)** | **0.83**  **(0.05)** | **19.6**  **(2.4)** | **1.4*10^3^**  **(0.1*10^3^)** | **9.0*10^3^**  **(2.8*10^3^)** | **3.1*10^2^**  **(3.3*10^2^)** | **0.16**  **(0.05)** | **0.73**  **(0.08)** |

Footnotes: #/vol.: number of bubble per unit volume; AV: average volume of bubbles; AAR (average aspect ratio): ratio between the shortest and the longest segment passing through the center of mass; AS (average sphericity): ratio of the diameter of the maximum inscribed sphere in a blob and the diameter of the sphere with the same volume as the blob; SSA (specific surface area): ratio between surface of the objects and their volume; IMC (integral of mean curvature): positive and negative value indicates the dominance of convex and concave objects (bubbles); EC (Euler characteristic): index of connectivity of the object network (bubbles), where positive and negative values are indicative of isolated *vs* connected/aggregates objects (bubbles). CD (connectivity density): number of redundant connections normalized to the total volume, with negative and positive values indicative of low and high connection; EI (elongation index): it measures the preferred orientation of a fabric, varying between 0 (no preferred orientation) and 1 (perfect preferred); IsI (isotropy index): similarity of fabric to a uniform distribution, changing between 0 (fully anisotropy) and 1 (perfect isotropy).

**Re**
